# Dissociation between Caloric and Video Head Impulse Tests in Dizziness Clinics

**DOI:** 10.3390/audiolres12040043

**Published:** 2022-08-08

**Authors:** Sofia Waissbluth, Valeria Sepúlveda

**Affiliations:** Department of Otolaryngology, Pontificia Universidad Católica de Chile, Santiago 8330033, Chile

**Keywords:** caloric test, head impulse test, Ménière’s disease, vestibular neuritis, vestibular migraine, vestibular schwannoma

## Abstract

Vestibular assessment tests such as the video head impulse test (vHIT) for the horizontal semicircular canal, and caloric test (Cal), both evaluate horizontal canal function. One would assume that the outcomes for these tests should lead to concordant results, yet several studies have suggested that dissociation can occur in certain pathological conditions. As this topic remains inconclusive, this review aims to analyze the scientific evidence regarding the patterns of hypofunction observed in vHIT and Cal in different otoneurological diseases. A comprehensive review of the literature regarding dissociation between these tests in common neurotological diseases was carried out. Articles were analyzed when data for vHIT and Cal were described in a way that it was possible to calculate discordance rates; both retrospective and prospective studies were analyzed. In this review, the discordance rates were as follows: 56% in Ménière’s disease, 51.5% in vestibular migraine, 37.2% in vestibular schwannoma, and 20.8% in vestibular neuritis. These results highlight the benefit of using both Cal and vHIT, and that they are complementary tests.

## 1. Introduction

As technology has evolved tremendously with regards to medical testing, it is now possible to evaluate all six semicircular canals independently using the video head impulse test (vHIT), since its development in 2009 [1,2,3]. Previously, caloric testing (Cal) was considered the gold standard for detecting unilateral hypofunction of the horizontal semicircular canal [4] through low frequency stimuli, around 0.002–0.004 Hz [5], hence, indirectly evaluating the superior vestibular nerve. Rotary chair testing also evaluates horizontal semicircular canal function, using mid-frequency stimuli, and is considered a useful tool for detecting bilateral vestibular losses [6,7].

Because the vHIT for the horizontal semicircular canal and Cal both evaluate the horizontal canal function, one would assume that the outcomes for these tests should lead to concordant results, yet several studies have suggested that dissociation can occur in certain pathological conditions [8,9,10,11,12,13,14,15,16]. The cristae ampullaris have two types of hair cells, type I and type II; they exhibit different receptor-afferent systems with distinct sensitivities. Type I hair cells synapse on flask-shaped calyx afferent fibers, which have high sensitivity to linear and angular forces, while type II hair cells synapse on bouton afferent fibers, which have low sensitivity to linear or angular forces [17]. They also differ in their localization on the cristae ampullaris. Both types are found throughout the cristae, both in the central and peripheral zones, yet around 70% of the hair cells are type I in the central zone, and approximately 50% in the peripheral zone [18]. It is then logical to think that the cristae probably respond differently to stimuli of different intensity. Lee et al. recently analyzed Cal and horizontal canal vHIT results in 893 consecutive patients, and they concluded that patients with abnormal Cal and normal vHIT mostly had peripheral lesions, while central lesions were likely to underlie those with abnormal vHIT and normal Cal [19].

As this topic remains inconclusive, we decided to analyze the scientific evidence regarding the patterns of hypofunction observed in vHIT and Cal in different otoneurological diseases. We conducted a comprehensive review of the literature regarding dissociation between these tests in common neurotological diseases. Articles were analyzed when data for vHIT (horizontal canal) and Cal were described in a way that it was possible to calculate discordance rates.

We performed a literature search using the PubMed database; only English language articles were reviewed. We employed the following PICOT framework to obtain the relevant articles:

- Population: Patients presenting dizziness; acute, chronic or episodic. Central etiologies, small sample size, and disorders with too few cases were not included.
- Intervention: Evaluation with Cal and vHIT.
- Comparison group: Comparison of dissociated results per disease between studies.
- Outcome of interest: Cal and vHIT results described in a way that it was possible to calculate discordance rates per disease. Caloric paresis for the Cal, and low gains and presence of saccades for the vHIT.
- Time: inpatients, outpatients.
- As a result of this search, we obtained 18 articles that evaluated patients with Ménière’s disease, vestibular neuritis, vestibular migraine and vestibular schwannoma.

## 2. Ménière’s Disease

Ménière’s disease (MD) is a clinical syndrome, without a clear etiology and, for now, is considered a multifactorial disorder. It presents clinically as a spontaneous episodic vestibular syndrome with recurrent vertigo attacks, and fluctuating hearing loss which over time becomes progressive with irreversible hearing loss. The disorder is quite variable in number and intensity of attacks, progression in time, and staging. Initially, patients can present with episodes of sudden sensorineural hearing loss that may be completely reversible, and without vertigo. Classically, the initial stages of the disease are associated with fluctuating tinnitus, aural fullness, hearing loss and vertigo attacks lasting between 20 min to 12 h [20], most commonly 20 min to two hours [21]. Because of the fluctuating nature of the disease and unpredictable pattern of audiovestibular damage, only audiometry demonstrating low- to medium-frequency sensorineural hearing loss is considered a diagnostic criterion for definitive MD [20]. Significant efforts have been made to understand why these patients have vertigo attacks, and numerous studies have been published with regards to vHIT, Cal, electrocochleography and vestibular evoked myogenic potentials in these patients, both during attacks and between attacks [22,23,24,25].

When considering studies with data regarding vHIT and Cal in patients diagnosed with MD, dissociation for these tests has been reported in 30–91%; most commonly normal gains on vHIT and canal paresis on Cal [9,26,27,28,29,30,31,32,33,34,35,36,37,38]. These results may vary according to the active or inactive phase of the disease. During unilateral MD attacks (active phase), both tests can be normal in 10–38%, both abnormal in 2–40%, low gains on vHIT and normal Cal in 18–30%, and normal gains on vHIT and hypofunction on Cal in 20–60% [26,28,30,36]. While in the inactive phase, 10–49% of patients have been reported to have abnormal findings in both tests, and 35–82% of the cases presented dissociation, where 9–18% had low gains on vHIT and normal response on Cal while 18–74% had normal gains on vHIT and hypofunction on Cal [9,26,29,30,31,32,33,34,35,37]. There is considerable variability for both test results, and their concordance rates. 

Limviriyakul et al. prospectively evaluated 51 patients with definitive Ménière’s disease with a median of 3.6 years for the duration of the disease; most patients had an active disease (78.8%). Overall, they observed that 76.5% had canal paresis on Cal, and an abnormal VOR gain in at least one canal in 47.1% of cases. When only analyzing the horizontal canal gain vs Cal, 56.9% had discordant results; mostly Cal paresis with normal gain on vHIT (*n* = 23/51). Interestingly, of the 39 patients with paresis on Cal, they report abnormal vHIT results for the unaffected side in fifteen patients (38.5%) [26]. Similarly, Shugyo et al. report canal paresis in 78.9% of cases with MD on Cal [35]. Yilmaz et al. report canal paresis in 66.1%, and of these patients, 41% also had abnormal gain of the lateral canal on vHIT. They also report overt saccades in 30% of the cases studied, and covert saccades in 7%, while 63% of MD patients did not exhibit any saccades [31].

Oliveira et al. evaluated 32 patients with definite MD with a mean duration of the disease of 3 years; seven patients had bilateral and 25 unilateral MD. They observed in symptomatic ears (*n* = 39) and asymptomatic ears (*n* = 25) that 56.4% (22/39) and 36% (9/25) had hypofunction on Cal, and that 25.6% (10/39) and 4% (1/25) had decreased VOR gain(s) in at least one semicircular canal, respectively. When analyzing vHIT vs. Cal, 43.6% (17/39) of the symptomatic ears had dissociation between tests with normal gains on vHIT and hypofunction on Cal and both tests abnormal in 12.8% (5/39); while in asymptomatic ears, 32% (8/29) had normal gains on vHIT with Cal paresis and 4% (1/25) had both tests altered [27]. Lee et al. analyzed the dissociated results between vHIT and Cal of patients with dizziness (*n* = 132). In the subgroup of patients diagnosed with MD (75/132), they found that 62 patients had normal VOR gains and canal paresis, and seven had low gains on vHIT and normal Cal [19]. McCaslin et al. also reported three cases of dissociated results in MD patients, all of them had normal gains on vHIT with canal paresis [8].

Endolymphatic hydrops has been linked to MD, although it is not a pathognomonic finding since hydrops has also been detected in asymptomatic patients, and in other otoneurological disorders. Endolymphatic hydrops leads to an enlargement of the membranous duct in the labyrinths of patients with MD. Based on the hydrostatic temperature dissipation hypothesis, an increase in the cross-sectional radius of the semicircular duct would have an insignificant effect on the response of the canal to angular acceleration stimulation, hence would not affect vHIT gains, yet it would cause a reduced response to caloric stimulation [9]. Therefore, hydropic expansion of the vestibular labyrinth, and not vestibular hypofunction could be the explanation for reduced caloric responses with normal vHIT gains in MD [39].

Semicircular canal plugging experiments have shown that because of compression, the sensitivity to low stimulus frequencies is attenuated, but not for high frequency stimuli. It has also been reported that distention of the membranous canal wall generated by utricular indentation causes transmembrane pressure gradients that allow flow into the region located between the ampulla and the canal plug [40]. Compression of the horizontal canal duct would affect low frequency responses, such as in Cal. Hence, this could also be a reason for discordance in MD.

Overall, it appears that around half of the patients with MD will have discordant results for vHIT and Cal (Table 1).

## 3. Vestibular Neuritis

Vestibular neuritis (VN) is the most common cause of acute vestibular syndrome, presenting with a sudden onset of vertigo that lasts longer than 24 hours and is associated with nausea, vomiting and head motion intolerance [41]. It most commonly affects exclusively the superior vestibular nerve [42,43], or in combination with the inferior nerve [44,45]. Isolated inferior vestibular nerve VN is rare. Temporal bone histological analysis has shown degeneration of peripheral vestibular nerve fibers and of the peripheral receptors’ neuroepithelium, most commonly the lateral canal ampulla and utricle [46]. Since most cases involve the superior vestibular nerve, which innervates the anterior canal, horizontal canal, and the utricle; testing for the horizontal canal with vHIT and Cal should provide comparable results. Various studies have described vHIT abnormalities for the horizontal canal in acute cases of VN with vHIT testing, varying from 91.4 to 97.7% [42,43,45]. 

During the acute phase of VN, it has been reported that 78–95% of patients have low gains on vHIT and Cal unilateral hypofunction [13,47], and that 95% present overt saccades, and 80% covert saccades [13]; only 5–10% had dissociated results, most commonly normal gains on vHIT and hypofunction on Cal [30,32,34,35,47]. In non-acute VN, it was reported that 21–80% have both tests that are normal, 25–67% have abnormal vHIT and Cal results, and 9–50% have normal gains on vHIT and hypofunction on Cal [11,30,33,47,48]. At this stage, 55% can exhibit overt saccades, and 65%, covert saccades [13]. Lee et al. analyzed the dissociated results between vHIT and Cal of patients with dizziness (*n* = 132). In the subgroup of patients diagnosed with VN (36/132), they found that 29 patients had normal VOR gains and canal paresis on Cal, and seven had low gains on vHIT and normal Cal [19]. Redondo-Martinez et al. suggest that vHIT gains recover at a faster rate than Cal unilateral weakness, hence, it is possible to obtain Cal hypofunction even in compensated VN while the vHIT may be normal [13]. Similar results were seen by Bartolomeo et al. [47]. While dissociation of vHIT and Cal has been observed for VN, both acute and non-acute, it is uncommon and seen in about 20% of cases, overall. Differences could be due to various factors, including the etiology and severity of the VN as well as central compensation strategies (Table 2). The fact is, Cal and vHIT are complementary tests, because they assess different responses from the crista ampullaris [48].

## 4. Vestibular Migraine

Vestibular migraine (VM) is the most common cause of episodic vestibular syndrome worldwide [49]. The term “vestibular migraine” was first introduced in 1999 [50], and in 2012, diagnostic criteria were published [51]. The diagnosis is based on the clinical manifestations of headache and vestibular symptoms, and clinical tests of vestibular function are not required [51]. There are no pathognomonic clinical signs or tests that confirm its diagnosis. In addition, patients report a wide spectrum of manifestations and test results, and there can be clinical overlap with other otoneurological conditions [52,53]. Research efforts have focused on its pathophysiology and on potential markers for this condition [54]. To date, there is extensive variability on test findings that may result from ictal/interictal testing, and duration of the condition [31,55,56,57,58]. It appears that Cal is more commonly abnormal in VM vs vHIT [30,31,36,59,60,61,62], and that patients with abnormal gains on VHIT frequently have hypofunction on Cal, which is more common than the other way around [60]. Moreover, it is not unusual to observe corrective saccades with normal gains on vHIT [63]. Directional preponderance on Cal has also been described as approximately 8–15% for VM [31,55,64]. 

In articles reporting dissociation between vHIT and Cal for VM patients (Table 3), most patients had normal gains on vHIT and hypofunction on Cal [30,31]. Mahringer and Rambold evaluated 16 patients with caloric hypofunction, and only two of these patients had abnormal gains on vHIT, hence 14/16 were discordant [30].

Yilmaz et al. reported that 34% of VM patients had hypofunction on Cal and that 18% had abnormal vHIT gains. Discordant results were seen in 40% (*n* = 20/50). They also report overt saccades in 8%, and covert saccades in 2%, while 90% of VM patients did not exhibit any saccades [31]. When taking these two studies into account, discordant results were observed in 51.5% of patients with VM (*n* = 34/66). Hannigan et al., on the other hand, is somewhat of an outlier; they retrospectively reviewed 140 cases of VM and reported that 139 had both normal Cal and vHIT results, and only one patient had Cal hypofunction [34]. 

## 5. Vestibular Schwannoma

A vestibular schwannoma (VS) is a benign tumor derived from Schwann cells, which arises from the vestibulocochlear nerve, and accounts for 85% of tumors in the cerebellopontine angle [65]. Although the most commonly described clinical presentation is progressive unilateral hearing loss and tinnitus [65], 40–50% of patients can present with vestibular dysfunction [66], and greater risk of postural instability and canal paresis has been associated with larger tumor size [67]. It can originate from the superior vestibular nerve, inferior vestibular nerve, cochlear nerve or facial nerve, but it most commonly arises from the inferior vestibular nerve [68,69]. In studies evaluating vHIT and Cal in VS, and that presented data that could be evaluated for discordance, we observed that abnormal Cal was more common than abnormal gains on vHIT in all studies, and that discordant results were seen in about a third of cases (Table 4).

Concordance of normal vHIT and Cal responses was reported in 7–30%, and both abnormal vHIT and Cal responses in 33–66% [14,34,70,71]. Tranter-Entwistle et al. prospectively evaluated 30 patients with unilateral VS. They found that 40% (12/30) of patients had dissociated results between vHIT and Cal, 36.7% (11/30) had normal VOR gains with hypofunction on Cal and 3.3% (1/30) had low gains on vHIT and normal Cal responses, while 30% of the patients had abnormal results in both tests and the other 30% had normal results [14]. Hannigan et al. reported 33.3% cases of normal gains with canal paresis in patients with VS [34]. West et al. reported 30.5% of dissociation with normal VOR gains and hypofunction on Cal. They also evaluated the presence or absence of saccades, 36% exhibited overt saccades, 3% covert and 34% both overt and covert saccades, while 27% did not exhibit any saccades. Presence of saccades was associated with a lower gain. Moreover, they measured the tumor size using magnetic resonance imaging, and found a correlation between tumor size and mean unilateral hypofunction on Cal [70]. Blödow et al. reported the greatest percentage of discordance for 69 cases of VS; discordance of 42% [71].

Interestingly, Brown et al. describe abnormal findings on Cal in 63%, and abnormal gains on the ipsilateral side in 31.4% on vHIT testing for 51 patients with VS. They also report that caloric weakness was associated with tumor size, and that there were no cases with abnormal vHIT and normal Cal [72]. It appears that Cal is more sensitive to detect vestibular abnormalities in patients with VS.

## 6. Limitations

We encountered variability with regards to the different criteria used to determine hypofunction on Cal. Most commonly, >25% of caloric paresis according to Jongkee’s formula was employed; however, some authors used >20% or even >30%. As for vHIT testing, an abnormal gain for the horizontal canal was predominantly considered as <0.8 or <0.79, and associated saccades were criteria for certain studies as well. Uncommonly, gain asymmetry was considered. Both retrospective and prospective studies were analyzed. Retrospective studies do have certain limitations, and this should be taken into consideration when reviewing this data. Another important aspect is timing. Only eight articles clearly reported that both tests were performed on the same day. This is relevant because patients with MD and VM have episodic vertigo, and testing at different time points could mislead the clinicians when trying to assess discordance rates. In addition, for VN, signs and symptoms will dissipate with time, hence testing the same day is the ideal scenario.

## 7. Conclusions

A discordant result between Cal and vHIT is not an uncommon finding in dizziness clinics. While both tests can measure unilateral weakness for the horizontal semicircular canal, it is known that both these tests demonstrate different responses to crista ampullaris stimulation. Other explanations have also been suggested as to why results may be discordant. For instance, whether high and/or low frequency vestibular nerve fibers are being affected, hence, frequency-dependent vestibular dysfunction and whether their rates of recovery vary, endolymphatic fluid retention, contributions of both peripheral and central vestibular deficits, ictal vs interictal testing, acute vs non-acute testing, and central compensation mechanisms. In this review, the discordance rates were as follows: 56% in Ménière’s disease, 51.5% in vestibular migraine, 37.2% in vestibular schwannoma, and 20.8% in vestibular neuritis. These results highlight the benefit of using both Cal and vHIT, and that they are complementary. It also displays that the vestibular system is complex and further research is needed to understand frequency-dependent damage and testing.

## Figures and Tables

**Table 1:** Ménière’s disease.

Study	Study Type	*n*	Both Tests				Caloric Test		vHIT	
			Both Normal	Both Abnormal	Dissociation	*n* Dissoc/*n* Total	Abnormality Definition (Unilateral Weakness)	Abnormal	Abnormality Definition (Unilateral Weakness)	Abnormal (Hor SCC)
**Young et al. 2021 [36]**	Prospective	50	19	1	30	30/50	CP ≥ 25%	31	gain < 0.8	1
**Sanyelbhaa et al. 2021 [37]**	Prospective	60	16	11	33	33/60	CP ≥ 25%	33 *	gain < 0.8	22
**Yilmaz et al. 2021 [31]**	Prospective	59	13	16	30	30/59	CP > 25%	39 (+4 had DP)	gain < 0.79	23
**Hannigan et al. 2021 [34]**	Retrospective	73	25	21	27	27/73	CP ≥30%	48	gain < 0.8	21
**Limviriyakul et al. 2020 [26]**	Prospective	51	4	21	29	29/51	CP > 25%	39	gain < 0.8	24
**Kitano et al. 2020 [33]**	Prospective	20	4	2	14	14/20	SPV response < 10°/s	16	gain < 0.8	2
**Shugyo et al. 2020 [35]**	Prospective	19	4	2	13	13/19	SPV < 20°/s	15	gain < 0.8	2
**Rubin et al. 2018 [38]**	Prospective	37	3	0	34	34/37	CP ≥ 20%	34	gain < 0.78	0
**Lee et al. 2017 [28]**	Retrospective	16 (11)	3	2	7	7/11	CP > 25%	7/11	gain < 0.8	12 **
**Cordero-Yanza et al. 2017 [29]**	Retrospective	88	28	19	41	41/88	CP > 20%	59	gain < 0.8	58
**Jung et al. 2017 [32]**	Retrospective	76	12	26	38	38/76	CP > 25%	55	gain < 0.8 with corrective saccade	31
**McGarvie et al. 2015 [9]**	Retrospective	22	0	4	18	18/22	CP > 25%	22	gain < 0.79	4
**Mahringer & Rambold 2014 [30]**	Retrospective	19	0	5	14	14/19	CP ≥ 25%	19	gain < 0.8	5
**Summary**						**56% (328/585)**				

CP: caloric paresis, DP: directional preponderance, SPV: slow phase velocity. * Unilateral: 24, Bilateral: 9, ** Unilateral: 7, Bilateral: 5.

**Table 2:** Vestibular neuritis.

Study	Study Type	*n*	Both Tests				Caloric Test		vHIT	
			Both Normal	Both Abnormal	Dissociation	*n* Dissoc/*n* Total	Abnormality Definition (Unilateral Weakness)	Abnormal	Abnormality Definition (Unilateral Weakness)	Abnormal (Hor SCC)
**Hannigan et al. 2021 [34]**	Retrospective	31	0	26	5	5/31	CP ≥ 30%	31	gain < 0.8	26
**Mekki et al. 2021 [48]**	Prospective	24	5	6	13	13/24	CP ≥ 20%	18	gain < 0.8 or gain asymmetry > 8%	7
**Kitano et al. 2020 [33]**	Prospective	10	8	0	2	2/10	SPV response < 10°/s	2	gain < 0.8	0
**Shugyo et al. 2020 [35]**	Prospective	11	1	9	1	1/11	SPV < 20°/s	9	gain < 0.8	10
**Jung et al. 2017 [32]**	Retrospective	19	0	18	1	1/19	CP ≥ 25%	19	gain < 0.8 with corrective saccade	18
**Bartolomeo et al. 2014 [47]**	Prospective	29	Acute:			10/58	CP >30%	Acute: 29	gain < 0.8	Acute: 26
			0	26	3					
			Follow-up:					Follow-up: 20		Follow-up: 13
			9	13	7					
**Mahringer & Rambold 2014 [30]**	Retrospective	29	Acute:			13/59	CP ≥ 25%	Acute: 29	gain < 0.8	Acute: 26
			0	26	3					
		30	Non-acute:					Non-acute: 30		Non-acute: 20
			0	20	10					
**Summary**						**21.2%** **(45/212)**				

CP: caloric paresis, SPV: slow phase velocity.

**Table 3:** Vestibular migraine.

Study	Study Type	*n*	Both Tests				Caloric Test		vHIT	
			Both Normal	Both Abnormal	Dissociation	*n* Dissoc/*n* Total	Abnormality Definition (Unilateral Weakness)	Abnormal	Abnormality Definition(Unilateral Weakness)	Abnormal (Hor SCC)
**Yilmaz et al. 2021 [31]**	Retrospective	50	27	3	20	20/50	CP >25%	17 (+4 had DP)	gain < 0.79	9
**Hannigan et al. 2021 [34]**	Retrospective	140	139	0	1	1/140	CP ≥ 30%	1	gain < 0.8	0
**Mahringer & Rambold 2014 [30]**	Retrospective	16	0	2	14	14/16	CP ≥ 25%	Acute: 2	gain< 0.8	Acute: 0
**Shugyo et al. 2020 [35]**	Prospective	11	1	9	1	1/11	SPV < 20°/s	Non-acute: 16	gain < 0.8	Non-acute: 2
**Summary**						**16.9%** **(35/206)**				

CP: caloric paresis, DP: directional preponderance.

**Table 4:** Vestibular schwannoma.

Study	Study Type	*n*	Both Tests				Caloric Test		vHIT	
			Both Normal	Both Abnormal	Dissociation	*n* Dissoc/*n* Total	Abnormality Definition (Unilateral Weakness)	Abnormal	Abnormality Definition (Unilateral Weakness)	Abnormal (Hor Scc)
**West et al. 2020 [70]**	Retrospective	59	4	37	18	18/59	CP > 25%	55	gain < 0.7 or asymmetry ratio > 8.5%	37 *
**Hannigan et al. 2021 [34]**	Retrospective	6	0	4	2	2/6	CP ≥ 30%	6	gain < 0.8	4
**Tranter-Entwistle et al. 2016 [14]**	Prospective	30	9	9	12	12/30	CP > 25%	20	gain < 0.79	10
**Blödow et al. 2014 [50]**	Retrospective	69	17	23	29	29/69	CP > 25%	50	gain < 0.79	25
**Summary**						**37.2%** **(61/164)**				

CP: caloric paresis, * 35 cases on the tumor side and 2 cases on the non-tumor side.

## Data Availability

Not applicable.
